# Association between time in therapeutic range of tacrolimus blood concentration and acute rejection within the first three months after lung transplantation

**DOI:** 10.1186/s40780-022-00256-9

**Published:** 2022-10-01

**Authors:** Yoshiki Katada, Shunsaku Nakagawa, Kotaro Itohara, Takuya Suzuki, Ryota Kato, Hiroki Endo, Mitsuhiro Sugimoto, Atsushi Yonezawa, Takayuki Nakagawa, Akihiro Ohsumi, Daisuke Nakajima, Hiroshi Date, Tomohiro Terada

**Affiliations:** 1 Department of Clinical Pharmacology and Therapeutics, Kyoto University Hospital, 54 Kawahara-cho, Shogoin, Kyoto, Sakyo-ku 606-8507 Japan; 2 Department of Thoracic Surgery, Kyoto University Graduate School of Medicine, 54 Kawahara-cho, Shogoin, Kyoto, Sakyo-ku 606-8507 Japan

**Keywords:** Tacrolimus, Time in therapeutic range, Lung transplantation, Acute rejection, Therapeutic drug monitoring

## Abstract

**Background:**

Tacrolimus is a key drug in immunosuppressive therapy following lung transplantation. The blood tacrolimus levels are likely to fluctuate in the early postoperative period, and failure to maintain the tacrolimus trough level in target ranges is a risk factor for rejection. However, there is little information about the relationship between the time in therapeutic range (TTR) of the tacrolimus trough level (tacrolimus TTR) and clinical outcomes. This study aimed to evaluate the association between tacrolimus TTR and acute rejection (AR) within the first three months after lung transplantation.

**Methods:**

This was a retrospective study of patients who underwent lung transplantation at a single center. The target tacrolimus trough levels were 10–15 ng/mL, and tacrolimus TTR was calculated using the Rosendaal method. The cut-off value of the tacrolimus TTR was estimated by receiver operating characteristic analysis based on AR.

**Results:**

The study included 90 patients. AR was observed in 26 patients. In this study, ‘‘early-AR’’ was defined as any AR within 2 weeks post-transplant (*n* = 22) and ‘‘late-AR’’ was defined as any AR after 1-month post-transplant (*n* = 4). For early AR, the relationship between tacrolimus TTR and the onset of AR was examined. There were no differences in the tacrolimus TTR between the early-AR group and non-AR group (35.7 ± 22.4 vs 31.5 ± 19.9%, *P* = 0.416). For late-AR, the relationship with tacrolimus TTR was examined every 10 d. The tacrolimus TTR during postoperative days (POD) 21–30 and POD 31–onset was significantly lower in the late-AR group than the no-AR group (50.0 ± 7.1 vs. 71.8 ± 18.0% and 37.0 ± 26.6 vs. 68.9 ± 31.5%, *P* < 0.05, respectively). The cutoff value of the tacrolimus TTR during POD 21–30 was estimated as 55.0%.

**Conclusions:**

Our findings suggest that a lower tacrolimus TTR is a predictor of late AR. A tacrolimus TTR of 55% or higher is necessary to reduce the risk of AR during this period after lung transplantation.

## Background

Lung transplantation has been established as a therapeutic option for patients with end-stage lung disease [1]. To prevent rejection after lung transplantation, a triple-drug combination of calcineurin inhibitors, antiproliferative agents, and corticosteroids is commonly prescribed as a maintenance immunosuppression regimen [2]. Calcineurin inhibitors, tacrolimus, and cyclosporine are the key drugs in this regimen. Tacrolimus has been suggested to show better outcomes than cyclosporine in lung transplantation [3], but has large inter- and intra-individual variability in its pharmacokinetics and a narrow therapeutic range of blood concentration [4]. Therefore, tacrolimus requires routine therapeutic drug monitoring (TDM) to minimize the risk of side effects and maximize its therapeutic efficacy. The target range of tacrolimus trough concentration for the first three months after lung transplantation is established at 10–15 ng/mL [5]. Failure to maintain the tacrolimus trough level within this target range is a risk factor for acute and chronic rejection after lung transplantation [6].

In general, for drugs whose pharmacological effects are concentration-dependent, the length of time for which the blood concentrations are in the therapeutic range is related to efficacy. For example, the effect of the anticoagulant, warfarin, is concentration dependent. It is well-known that the duration ratio in the target therapeutic range of prothrombin time (time in therapeutic range (TTR), %), a marker of warfarin blood levels, predicts efficacy and adverse events [7]. Therefore, the TTR of prothrombin time has been used as a validated measure to assess effective warfarin therapy. As for tacrolimus, variations in dosage or blood concentrations can lead to therapeutic failure or adverse reactions. Therefore, the TTR of blood tacrolimus concentration (tacrolimus TTR) may be useful in managing tacrolimus therapy. Recently, an association between tacrolimus TTR and rates of donor-specific antibody production or acute rejection (AR) in lung and kidney transplant patients was reported [8, 9]. Thus, the tacrolimus TTR may be a potential prognostic indicator of clinical outcomes after organ transplantation.

The incidence of AR after lung transplantation is highest in the first three months and decreases with time [10]. In addition, the blood tacrolimus levels are likely to fluctuate in the early postoperative period because of hemodynamic instability, the need for blood transfusions, and the occurrence of systemic inflammation [11]. Therefore, it is necessary to investigate the relationship between clinical outcomes and tacrolimus TTR in the early phase after lung transplantation. The purpose of this study was to evaluate the association between tacrolimus TTR and AR within the first three months after lung transplantation. We hypothesized that a lower tacrolimus TTR would be associated with an increased risk of AR in lung transplant patients.

## Methods

### Patients population

Patients who underwent lung transplantation at Kyoto University Hospital between January 2016 and December 2020 were retrospectively analyzed. Patients who concomitantly used basiliximab or changed tacrolimus to cyclosporine were excluded from this analysis because the target range of tacrolimus trough concentration was different from that of other patients. Electronic medical records were reviewed and pertinent data were retrieved. The collected data included patient demographics, medical history, transplantation details, and medications. This study was approved by the Ethics Committee of Kyoto University Graduate School and Faculty of Medicine (R0545-2). The Ethics Committee waived the need for informed consent since this was an observational study using existing data.

### Immunosuppression regimen

A combination of tacrolimus, mycophenolate mofetil, and corticosteroids was used in all patients. Tacrolimus was initiated at the discretion of the transplant team. Mycophenolate mofetil was orally administered at 1500 mg/day, and the dosage was reduced in cases such as intolerable gastrointestinal side effects or leukopenia. Corticosteroid therapy was initiated with intravenous methylprednisolone at 125 mg/day for 3 d after transplantation and reduced to a stable dose of oral prednisolone (0.4 mg/kg) using a weekly weaning regimen.

The measurements of blood tacrolimus levels were usually performed daily during intensive care unit stay and then twice or thrice weekly after transfer to the general ward until discharge. Micafungin was initiated in the perioperative period, and itraconazole oral solution was administered when the patients tolerated the oral intake. The concentrations of itraconazole and its major metabolite hydroxyitraconazole were measured in all cases. After the sum of the serum itraconazole and hydroxyitraconazole concentrations reached 750 ng/mL, micafungin administration was stopped. After the initiation of itraconazole, the blood tacrolimus levels were measured at least daily until the tacrolimus trough concentration was stable. Tacrolimus levels in the blood were measured using a chemiluminescent immunoassay kit (ARCHITECT®, Abbott Japan, Chiba, Japan). Trough levels of tacrolimus were measured the morning prior to drug administration. The tacrolimus dosage was adjusted to trough concentrations of 10–15 ng/mL in the first three months after transplantation.

### Time in therapeutic range

The Rosendaal linear interpolation method was used to calculate tacrolimus TTR [12]. The tacrolimus TTR was calculated within the first three months after lung transplantation. The TTR was normalized as a percentage of tacrolimus administration every 10 d after lung transplantation. The mean tacrolimus trough concentration was calculated every 10 d.

### Study endpoints

The primary outcome was the clinical AR within the first three months after lung transplantation. The transplant physicians judged the AR based on radiographic and clinical findings without transbronchial lung biopsy. AR episodes were characterized by dyspnea, low-grade fever, leukocytosis, hypoxemia, and diffuse interstitial infiltrates on chest radiographs. Patients with AR were primarily treated with bolus doses of methylprednisolone and were closely monitored for various clinical signs. Rabbit anti-thymocyte globulin was administered in cases of hemodynamic compromise, persistent AR, or severe rejection. In this study, AR was defined as a suspected rejection that was improved after the treatment with methylprednisolone or rabbit-antithymocyte globulin.

### Statistical analysis

At our institution, 40% of patients developed AR within 5 years after lung transplantation [13]; however, the incidence within 3 months after transplantation remains unknown. There have been no studies evaluating tacrolimus TTR after lung transplantation in Japan. Therefore, we did not have enough information to perform a formal power calculation. Our institution conducts a limited yet the highest number of lung transplants in Japan, approximately 30 per year. Therefore, we included all recipients who underwent lung transplantation at our institution during the observation period. Relationships between AR and tacrolimus TTR, patient demographics, and clinical characteristics were analyzed using one-way ANOVA for continuous variables and the chi-square test or Fisher’s exact test for categorical variables. AR and tacrolimus TTR or trough concentrations were compared using Student’s *t*-test. A receiver operating characteristic (ROC) curve was constructed to determine the thresholds for the optimal tacrolimus TTR. All analyses were performed using GraphPad Prism (GraphPad Software version 9.2.0, Inc., San Diego, CA, USA). The alpha level of significance was set at *P* < 0.05.

## Results

**Fig. 1:**
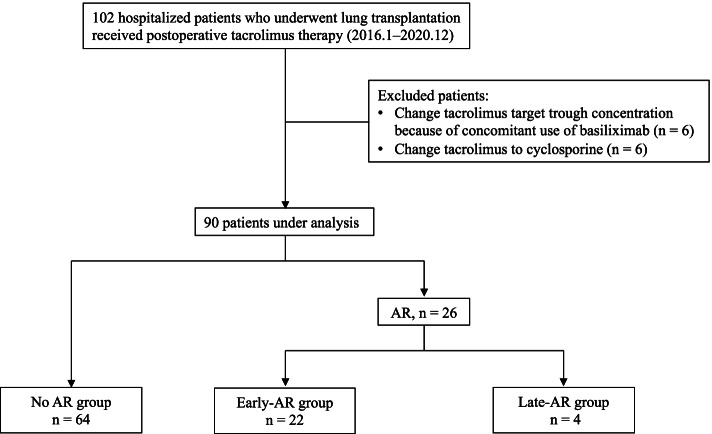
Flowchart of patient selection. AR: acute rejection

**Fig. 2:**
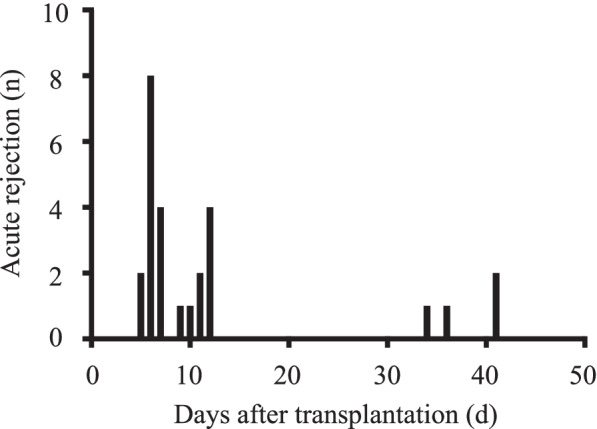
Time to event for acute rejection. The onset of AR is bimodal. Early AR was defined as any AR within 2 weeks post-transplant (*n* = 22), and late AR was defined as any AR after 1-month post-transplant (*n* = 4)

A flowchart of the patient selection process is shown in Fig. 1. During the study period, 102 patients underwent lung transplantation. Of these, 12 patients were excluded from the analysis because basiliximab was concomitantly administered, or tacrolimus was changed to cyclosporine. Hence, 90 patients were included in the study. AR was observed in 26 patients. The occurrence of AR according to the time post-transplantation is outlined in Fig. 2. The onset of AR was bimodal, with some occurring two weeks after transplantation and others occurring after 4 weeks (8.1 ± 2.5 d [*n* = 22] and 38.0 ± 3.1 d [*n* = 4], respectively). In this study, ‘‘early-AR’’ was defined as any AR within 2 weeks post-transplantation (*n* = 22) and ‘‘late-AR’’ was defined as any AR after 4 weeks post-transplantation (*n* = 4).

Patient demographics and clinical characteristics were compared among the following three groups: no AR group (*n* = 64), early AR group (*n* = 22), and late AR group (*n* = 4; Table 1). Significant differences were observed in length of stay in the intensive care unit (*P* = 0.009), length of hospital stay (from postoperative to discharge, *P* = 0.006), and the ratio of infection after lung transplantation (*P* = 0.014).

**Table 1:** Patients characteristics

	No AR group *n* = 64	Early-AR group *n* = 22	Late-AR group *n* = 4	*P*-value
Age (years), mean ± SD	40.4 ± 16.7	41.9 ± 12.7	38.5 ± 15.1	0.896
Sex (male/female)	28/36	13/9	0/4	0.079
Body weight (kg), mean ± SD	45.3 ± 13.3	50.8 ± 15.4	42.3 ± 9.2	0.227
Body mass index (kg/m^2^), mean ± SD	17.9 ± 3.3	19.4 ± 4.0	17.2 ± 2.8	0.195
Indication for transplant, n (%)				
Interstitial pneumonia	29 (45)	9 (41)	2 (50)	0.913
BO after HSCT	9 (14)	4 (18)	0 (0)	0.627
Pulmonary hypertension	6 (9)	1 (5)	1 (25)	0.403
Others	20 (32)	8 (36)	1 (25)	0.862
LT type (LDLT/DDLT)	16/48	7/15	1/3	0.820
LT type (Bilateral/Single)	41/23	19/3	3/1	0.140
Baseline aspartate aminotransferase (IU/L), mean ± SD	25.2 ± 17.9	19.2 ± 6.7	27.0 ± 7.7	0.283
Baseline alanine aminotransferase (IU/L), mean ± SD	18.0 ± 12.0	16.0 ± 7.9	23.8 ± 10.7	0.425
Baseline total bilirubin (mg/dL), mean ± SD	0.6 ± 0.3	0.7 ± 0.3	0.4 ± 0.1	0.266
Baseline serum creatinine (mg/dL), mean ± SD	0.6 ± 0.2	0.6 ± 0.2	0.7 ± 0.3	0.166
Baseline eGFR (mL/min/1.73m^2^), mean ± SD	143.2 ± 103.2	135.3 ± 172.9	113.6 ± 78.9	0.882
Baseline albumin (g/dL), mean ± SD	3.9 ± 0.5	3.9 ± 0.5	3.5 ± 0.7	0.217
With diabetes mellitus, n (%)	3 (5)	0 (0)	0 (0)	0.532
With dialysis, n (%)	3 (5)	0 (0)	0 (0)	0.532
Tacrolimus use before LT, n (%)	8 (13)	5 (23)	2 (50)	0.101
Anti HLA antibody ( +) before LT, n (%)	6 (9)	0 (0)	1 (25)	0.154
Operative time (h), mean ± SD	7.8 ± 2.9	9.5 ± 2.6	7.8 ± 2.4	0.069
Operative blood loss (mL), mean ± SD	2803.0 ± 4155.0	4765.2 ± 6445.7	2061.5 ± 1233.0	0.233
Length of ICU stay (days), mean ± SD	12.8 ± 7.7	18.4 ± 5.3	15.0 ± 6.8	0.009
Length of hospital stays from postoperative to discharge (days), mean ± SD	63.2 ± 27.9	85.9 ± 61.5	117.0 ± 41.9	0.006
Concomitant use of ITCZ, n (%)	63 (98)	21 (95)	4 (100)	0.682
Discontinuation of MMF, n (%)	6 (9)	2 (9)	0 (0)	0.814
Infection after LT, n (%)	10 (16)	5 (22)	3 (75)	0.014

Data are represented either as n (%) or mean ± standard deviation

*AR* Acute rejection, *BO* Bronchiolitis obliterans, *HSCT* Hematopoietic stem cell transplantation, *LT* Lung transplantation, *LDLT* Living-donor lung transplantation, *DDLT* Deceased-donor lung transplantation, *eGFR* estimated glomerular filtration rate, *HLA* Human leukocyte antigen, *ICU* Intensive care unit, *ITCZ* Itraconazole, *MMF* mycophenolate mofetil

We assumed an association between AR and tacrolimus TTR in the period preceding AR. Therefore, the tacrolimus TTR to AR onset in patients with early AR was compared with that on day 8 in patients who did not develop AR (Table 2). The results showed no significant difference in the values between the two groups (35.7 ± 22.4 % vs 31.5 ± 19.9 %, *P* = 0.416). For late AR, the relationship between AR and tacrolimus TTR every 10 d was investigated to determine which period of TTR was associated with AR (Table 3). The tacrolimus TTR during postoperative days 21–30 and postoperative days 31–onset was significantly lower in late-AR group than in no AR group (50.0 ± 7.1 % vs 71.8 ± 18.0 %, 37.0 ± 26.6 % vs 68.9 ± 31.5 %, *P* < 0.05, respectively). The relationship between tacrolimus trough concentration and early or late AR is shown in Tables 4 and 5. There was no correlation between the tacrolimus trough concentration and AR.

**Table 2:** Relationship between tacrolimus time in therapeutic range and early-acute rejection

	No AR group *n* = 64	Early-AR group *n* = 22	*P*-value
Period	Time in therapeutic range (%)		
POD: 1 ~ onset	31.5 ± 19.9	35.7 ± 22.4	0.416

The results are presented as the mean ± standard deviation. Onset in the No AR group was defined as the mean number of days of developing early-AR. *POD* Postoperative day, *AR* Acute rejection

**Table 3:** Relationship between tacrolimus time in therapeutic range and late-acute rejection

	No AR group *n* = 64	Late-AR group *n* = 4	*P*-value
Period	Time in therapeutic range (%)		
POD: 1 ~ onset	53.5 ± 14.7	43.0 ± 10.3	0.171
POD: 1 ~ 10	38.8 ± 17.3	31.3 ± 6.5	0.397
POD: 11 ~ 20	58.2 ± 24.4	52.5 ± 19.2	0.653
POD: 21 ~ 30	71.8 ± 18.0	50.0 ± 7.1	0.020
POD: 31 ~ onset	68.9 ± 31.5	37.0 ± 26.6	0.041

The results are presented as the mean ± standard deviation. Onset in the No AR group was defined as the mean number of days of late-AR development. *POD* Postoperative day, *AR* Acute rejection

**Table 4:** Relationship between tacrolimus trough concentration and early-acute rejection

	No AR group *n* = 64	Early-AR group *n* = 22	*P*-value
Period	Trough concentration (ng/mL)		
POD: 1 ~ onset	10.0 ± 2.6	10.8 ± 2.7	0.214

The results are presented as the mean ± standard deviation. Onset in the No AR group was defined as the mean number of days of developing early-AR. *POD* Postoperative day, *AR* Acute rejection

**Table 5:** Relationship between tacrolimus trough concentration and late-acute rejection

	No AR group *n* = 64	Late-AR group *n* = 4	*P*-value
Period	Trough concentration (ng/mL)		
POD: 1 ~ onset	12.4 ± 1.5	11.3 ± 0.3	0.138
POD: 1 ~ 10	10.7 ± 1.8	9.2 ± 0.6	0.116
POD: 11 ~ 20	12.8 ± 2.1	13.5 ± 1.0	0.522
POD: 21 ~ 30	13.1 ± 1.8	12.0 ± 1.2	0.195
POD: 31 ~ onset	12.9 ± 2.3	10.7 ± 2.0	0.073

The results are presented as the mean ± standard deviation. Onset in the No AR group was defined as the mean number of days of late-AR development. *POD* Postoperative day, *AR* Acute rejection

**Fig. 3:**
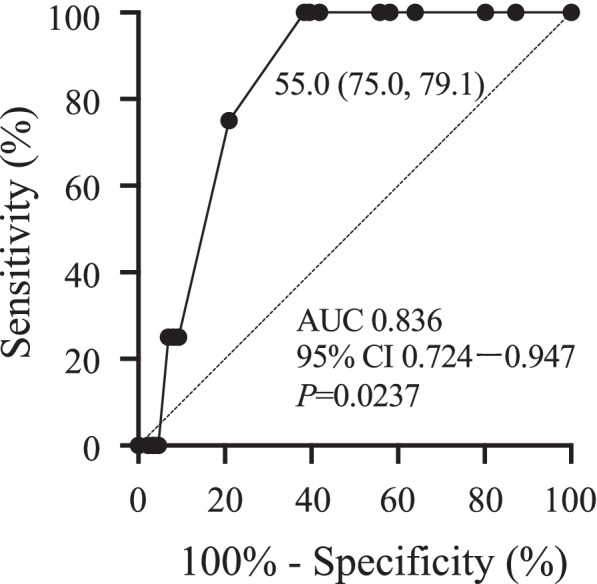
ROC curve analysis of the cut-off value of tacrolimus TTR during postoperative days 21–30 to prevent AR late-onset. 95% CI: 95% confidence interval, ROC: receiver operating characteristic, TTR: time in therapeutic range, AR: acute rejection

The threshold value of tacrolimus TTR during postoperative days 21–30 was evaluated. The cutoff value of the tacrolimus TTR during postoperative days 21–30 was estimated as 55.0 % (area under the curve = 0.836, 95% CI = 0.724–0.947, *P* = 0.0237; Fig. 3).

**Fig. 4:**
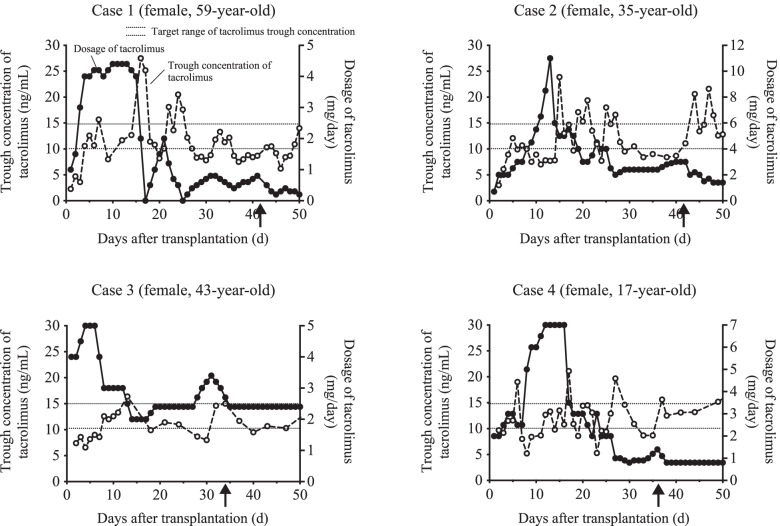
Time course of trough blood concentrations and dosage of tacrolimus in four cases that occurred late-AR. Closed and open circles show the dosage and trough concentration of tacrolimus, respectively. *X*-axis represents the number of days after transplantation. The arrow indicates when AR was observed

Figure 4 shows the time course of trough blood concentrations and dosages of tacrolimus in four late-AR cases. In all cases, there was a decrease in tacrolimus concentrations, which was lower than the target concentration range, before the onset of AR. In case #1, the pulmonary *Mycobacterium avium* complex disease was developed on postoperative day 29, and clarithromycin, amikacin, and cefmetazole were administered to treat this infection. In case #2, the patient did not comply with itraconazole oral solution when its administration began on postoperative day 25. In case #3, the patient had watery diarrhea due to cytomegalovirus colitis (5–10 times/day) from postoperative day 25, and ganciclovir was administered to treat this infection. In case #4, the antifungal prophylaxis was changed from itraconazole to micafungin due to suspected breakthrough fungal infection on postoperative day 30. Infection was observed in cases #1, #2, and #4. Antimicrobial therapy was administered from the day these infections were observed.

## Discussion

AR is a major complication from lung transplantation. In previous studies investigating the impact of tacrolimus TTR in renal transplant patients, the cut-off values in the first year after transplantation were estimated as 60% [8], 78% [14], and 40% [15]. These cutoff values are associated with AR [8], graft loss [8, 14, 15], de novo donor-specific antibody formation [8, 15], and patient death [14]. Similarly, in lung transplant patients, tacrolimus TTR < 30% was associated with AR and chronic lung allograft dysfunction in the first year after transplantation [9]. These researchers found that a higher tacrolimus TTR was associated with improved clinical outcomes, but the relationship between the occurrence of rejection and tacrolimus TTR in the early postoperative period is unknown. In this single-center study, we found an association between tacrolimus TTR and AR onset within three months in lung transplant patients. We found a significant correlation between tacrolimus-TTR and late-AR. The cut-off value of tacrolimus TTR for late-AR was estimated as 55%, which is different from that estimated in a previous study [9]. Possible reasons for this difference include: i) different observation periods used to calculate tacrolimus TTR in the two studies, ii) different target ranges of tacrolimus concentrations, and iii) the influence of race and unmeasured factors. Although further studies are needed to define the cut-off value of tacrolimus TTR, our results suggest that blood levels of tacrolimus should be strictly controlled even in the first three months after lung transplantation.

Tacrolimus exerts its effect through tertiary complexation with FK506 binding protein 12 and calcineurin, leading to decreased nuclear production of interleukin-2 and thereby reduced lymphocyte proliferation. This complex inhibits the activity of the enzyme calcineurin and thus, interrupts the calcium-dependent signal-transduction pathway in T cells [16]. Since the transplant recipients included in this study did not undergo transbronchial lung biopsy, it is unclear whether late-AR is acute cellular rejection or not. However, we consider that low tacrolimus TTR results in AR, as the clinical symptoms improved with steroid pulse implementation.

In this study, a decrease in tacrolimus TTR was observed 10 d before the onset of late-AR. This suggests that a decrease in tacrolimus TTR may influence/promote AR in lung transplant patients over a short period of time. Of the four patients with late-AR, three developed infections after transplantation. Since immunosuppressive therapy increases the risk of infection, tacrolimus dose reduction is usually considered when the infection is diagnosed. In these cases, it is possible that the tacrolimus blood levels were adjusted to the lower end of the target range. In addition, the antibiotic and antifungal doses were changed prior to the onset of AR, in these cases. Tacrolimus is metabolized by cytochrome P450 (CYP) 3A in the liver and small intestine [17]. Itraconazole and clarithromycin, CYP3A inhibitors, have a significant effect on the rate of tacrolimus absorption and metabolism, thus altering its concentration [18, 19]. Therefore, prescribing CYP3A inhibitors or changes in their doses may cause fluctuations in tacrolimus concentrations in cases with late-AR.

AR is considerably prevalent in the early post-transplant period. In a previous study, AR occurred mostly within the first postoperative month, observed in 26% of patients (63 of 241) [10]. In this study, the incidence of early-AR was 25% (23 of 92), which is comparable to the rate reported in the previous study. However, we could not find a significant correlation between AR and tacrolimus TTR within two weeks after transplantation. The tacrolimus TTR up to two postoperative weeks was lower than the tacrolimus TTR thereafter, suggesting that the pharmacokinetics of tacrolimus was not stable up to two postoperative weeks. Additionally, the rate of AR could be affected by other immunosuppressants such as methylprednisolone and mycophenolate mofetil. A previous study reported no correlation between tacrolimus TTR and AR in the first week after renal transplantation [20]. Another study reported that tacrolimus concentrations within 14 d after lung transplantation was significantly associated with acute kidney injury but not with AR [21]. In these previous studies, basiliximab and high-dose steroids were also used as immunosuppressants. This suggests that early-AR could be influenced by the concomitant use of immunosuppressive agents other than tacrolimus. In contrast, high tacrolimus concentrations have been reported to increase the risk of acute kidney injury even early after lung transplantation [22]. Taken together, the higher tacrolimus TTR in the early period after lung transplantation may be less important when used in combination with other immunosuppressants such as high-dose methylprednisolone and mycophenolate mofetil.

This study has several important limitations. First, this study had a limited sample size, which makes it difficult to examine the effects of potential confounders and perform subgroup analysis. Second, because this was a retrospective and non-randomized observational investigation, various unmeasured confounders may have resulted in a bias-derived outcome. Third, there was a variation in the measurement intervals of tacrolimus blood levels. Therefore, the estimated tacrolimus concentration may not have accurately reflected the real exposures. Fourth, AR is determined by clinical symptoms and steroid pulse implementation and not by transbronchial lung biopsy. Therefore, the potential for AR may not have been determined. In addition, it is unclear whether AR is acute cellular rejection or not.

## Conclusions

This study is the first to assess the effect of tacrolimus TTR in the early postoperative period of lung transplantation. Our findings suggest that a lower tacrolimus TTR is a predictor of AR four weeks after lung transplantation. A tacrolimus TTR of 55% or higher is necessary to reduce the risk of AR during this period.

## Data Availability

The datasets used and/or analyzed during the present study are available from the corresponding author upon reasonable request.

## Abbreviations

TDM: Therapeutic drug monitoring
TTR: Time in therapeutic range
AR: Acute rejection
ROC: Receiver operating characteristic
CYP: Cytochrome P450
